# The Breakthrough of Biosimilars: A Twist in the Narrative of Biological Therapy

**DOI:** 10.3390/biom9090410

**Published:** 2019-08-24

**Authors:** Eva Rahman Kabir, Shannon Sherwin Moreino, Mohammad Kawsar Sharif Siam

**Affiliations:** 1 Department of Pharmacy, BRAC University, Dhaka 1212, Bangladesh; 2 Department of Chemistry, University of Cambridge, Cambridge CB2 1EW, UK

**Keywords:** biosimilar, biologic, challenges, regulatory, immunogenicity, biosimilar market

## Abstract

The coming wave of patent expiries of first generation commercialized biotherapeutical drugs has seen the global market open its doors to close copies of these products. These near perfect substitutes, which are termed as “biosimilars”, do not need to undergo intense clinical trials for their approval. However, they are mandated to produce identical similarity from their reference biologics in terms of clinical safety and efficacy. As such, these biosimilar products promise to foster unprecedented access to a wide range of life-saving biologics. However, seeing this promise be fulfilled requires the development of biosimilars to be augmented with product trust, predictable regulatory frameworks, and sustainable policies. It is vital for healthcare and marketing professionals to understand the critical challenges surrounding biosimilar use and implement informed clinical and commercial decisions. A proper framework of pharmacovigilance, education, and scientific exchange for biologics and biosimilars would ensure a dramatic rise in healthcare access and market sustainability. This paper seeks to collate and review all relevant published intelligence of the health and business potential of biosimilars. In doing so, it provides a visualization of the essential steps that are required to be taken for global biosimilar acceptance.

## 1. Introduction

The rise and diversification of biologic drugs has witnessed several challenges and opportunities on its path to global acceptance [1]. With the current landscape of these products promising continuous growth, biological therapy is expanding the reach of medicine without restricting the research and development of small molecule drugs.

Biologic medications (biologics) are complex macromolecular drugs that are manufactured via living systems. The development of targeted biologics has revolutionized the treatment of several severe and chronic diseases [2]. The explosion in the evolution of these drugs have been instrumental in tackling treatment strategies with regard to cancer (monoclonal antibodies), autoimmune conditions, diabetes (human insulin), and anemia (erythropoietin substitutes) [3]. However, these brand name agents tend to be expensive, which prompts the need for more cost-effective solutions to come to the market.

With the increasing medical and commercial success of recombinant proteins and monoclonal antibodies (Appendix A1), a large number of pharmaceutical companies have strived to become key players in the biologics field. Furthermore, the range of biotherapeutic therapies have also since expanded to include nanobodies (Appendix A2), soluble receptors, fusion proteins, immuno-therapies (Appendix A3), synthetic vaccines, immunoconjugates (Appendix A4), modified proteins (glycosylated and pegylated), etc. These therapies have emerged as a result of the contributions of new technologies and an improved understanding of cell line production, protein identification, expression, and engineering [4].

Pharmaceutical companies are encouraged by several reasons to move to a biologics-oriented industry. Firstly, biologics possess the capability to bind with target sites that tend to be elusive or inaccessible to small molecule drugs [5]. This can be related with protein-protein interactions that involve flat surfaces with fewer charged regions. Secondly, biologics hold highly propitious commercial potential. Around $194 billion worth sales of biologics are at risk between the years 2017 and 2022 [6]. This signals the beginning of a second patent cliff era of blockbuster biologic drug products being challenged by biosimilars. Thirdly, there is a better overall economic return that is delivered by biologics as opposed to that of small molecule drugs [7].

As opposed to small molecule drugs, there are several complexities that are associated with the development and manufacturing processes of complex large molecular biologics. Biologics therapy is expensive and it places a substantial financial burden on the healthcare system. Furthermore, these high costs restrict the availability and accessibility of such drugs to only those who can afford them. Certain countries, such as Australia, have responded to these high costs by limiting the administration of biologics to only indications that receive reimbursement through their Pharmaceutical Benefits Scheme (PBS) [8]. However, issues, such as formulary inclusions, drug availability, and patient out-of-pocket costs still imply that access to biologics treatment still remain limited in several developing and even certain developed countries.

The looming expiry of patents [9] and limited biologics accessibility have, however, spelled big opportunity for another branch of medicine—biosimilars. The US Food and Drug Administration (US FDA) states that biosimilars are mandated to demonstrate high similarity to their licensed originator biologics in terms of their biochemical, immunological, safety, and biological properties [10]. It is vital that these substitutes for biologic drugs possess no clinically meaningful differences. Any variations present in the macromolecule are required to be restricted only within its clinically inactive components.

Biosimilar drugs hold the potential to penetrate the market by the year 2020 for a large number of important biologic drugs; having sales that aggregate over 40 billion euros [11]. The market for biosimilars has seen strong advancements in the United States and European countries. The market is also steadily gaining traction in countries with preexisting biologics manufacturing industries and enterprises [7]. A paper by Grant Thornton India states how an estimated 21 biologics with a market net worth $50 billion will lose patent (Appendix A5) protection in USA alone between the years 2009 and 2019 [12].

However, it is important to understand that biosimilars are not identifiable with generic versions of newly innovative drugs, and therefore do not dictate therapeutic equivalence by default. The term “generic” medication is utilized to identify chemical small molecule drugs that possess structural and therapeutic equivalence to a reference product (usually one whose patent period has reached expiration). On the other hand, biologics are harder to anatomically characterize. Biologics are a hundred to a thousand times larger in size than synthetic small molecule drugs. They possess several hundred amino acids that are biochemically combined in a definite sequence [13]. As a result, biologics tend to possess various secondary or tertiary structural structural and post translational modification variations. Structural differences brought about by glycation, oxidation, glycosylation, sulfide crosslinking, etc. could be present within the same batch of biologics. With these minor differences being very difficult to remove, highly similar biosimilar and reference biologics do not necessarily express the same clinical efficacy. There are several more challenges to the production of these products as compared to conventional generic drugs, mainly owing to their complex large molecular structure [14]. Process related or structural variations between a biosimilar and its reference biologic could lead to drastic changes in the effectiveness and safety profile in the biosimilar product. This expresses an even greater risk in the case for more complex biologics where the drug mechanisms may not be fully understood. Furthermore, variations in the age, sex, gender, etc. parameters of the targeted patient groups may lead to different responses to the same biotherapeutic utilized [15].

### Basic Concept

As defined by the US FDA, a biosimilar product is a biological product that is approved based on a showing that it is highly similar to an already approved biological product, which is known as a reference product, and that there are no clinically meaningful differences between the biologic product and the reference product in terms of safety, purity, and potency of the product [16]. These products are manufactured through defined and stringent regulatory processes after having undergone rigorous analytical, non-clinical, immunogenicity, and clinical comparative evaluations. Biosimilar products must be shown to have no clinically meaningful differences in terms of safety and effectiveness from the reference product. This means that these products are therefore not innovative in terms of indication, therapeutic target, administration route, or regimen. Table 1 shows the definition of biosimilars, as set by the World Health Organization (WHO), the European Medicines Agency (EMA), the United States Food & Drug Administration (US FDA), the Biologics and Genetic Therapies Directorate (BGTD) of Canada, the Pharmaceuticals and Medical Devices Agency (PMDA) of Japan, and the Therapeutic Goods Administration (TGA) of Australia.

#### Biosimilars or Not?

Biological products whose active ingredients or formulations are slightly modified with the aim of improving efficacy or ameliorating therapeutic regimen are separately distinguished as “biobetters” [17]. Such products may be improved through favourable pharmacodynamic or pharmacokinetic alterations (such as improving the potency or half-life of the reference biologic) without any change in the therapeutic attributes of the reference drug. However, if the biological product manufactured is innovative in therapeutic target, indication, or regimen, it is classified as an “innovative biologic”. Innovative biologics are usually superior to their reference counterparts.

Biological medicines are also differentiated based on their degree of conformity with globally recognized regulatory standards. Copies of originator biologics that have not undergone rigorous evaluations as biosimilars and have not met the strict regulatory requirements that were set by the WHO, EMA, or US FDA are termed as non-comparable biotherapeutic products (also known as intended copies, non-original biologics, or biomimics). These products tend to have a questionable similarity of API and are often licensed in countries that lack stringent regulatory pathways (or regulations distant from that of WHO, EMA or US FDA). However, studies have provided evidence of these products possessing considerable structural and clinical differences from their reference counterparts and pose a huge risk to patients within these countries [18,19,20]. Biomimics originated prior to the implementation of science-based regulatory pathways for the approval of biosimilars and they were only introduced in some countries. The basis for approval of these copies has not been clear, as they lack comparative studies to an appropriate reference product [21].

In certain cases, a pharmaceutical company may come to the decision to not pursue the biosimilar regulatory pathway, despite the apparent similarity with a biological already in the market. This may be due to commercial reasons or recommendations that were obtained from regulatory bodies. In such situations, these companies may choose to progress via a “stand alone application”. This application was conceptualized but not approved by the EMA [22], and it involves any biological manufacturing request that depends on its own manufacturer data for approval. An example of this can be seen with Eporatio—an erythropoietin that was approved by the European Commission in 2009 [23].

## 2. Regulatory Compliance

The manufacture of biologics in pharmaceutical industries began in the early 1980s [24]. However, their financial strain and the presence of a patent cliff (a steady decline in sales as the biologic nears the expiry of its patent) made the need for follow-on biologics inevitable. Several countries stringently regulate their biosimilar approval requirements, which ensures that they are scientifically aligned on accepted principles. The emergence of globally consistent approval processes would allow for an easier adoption of biosimilars and reduce the complexities of its regulatory landscape. However, several key inconsistencies are still present within the regulatory framework, such as the choice of reference product, extrapolation of indications, and comparability study designs [25].

Adopting globally approved regulatory standards would promote patient and clinician confidence in the prescription and consumption of biosimilar drugs, respectively. It is strongly recommended that manufacturers discuss the need for post biosimilar approval trials, registries, and pharmacovigilance activities with regulators. Such action could serve to eliminate any remaining uncertainty on biosimilarity obtained from the Phase I trial data collected. Drug regulatory authorities usually take such post approval commitment decisions for any newly designed biological or chemical drug products.

### 2.1. The Evolving Regulatory Landscape of Biosimilars

The European Medicines Agency was the first regulatory authority to set guidelines for biosimilars in 2005, a year before the first biosimilar was approved. The member states of the European Union (EU) hold the power of implementing any regulations with regard to the manufacture, development, and authorization of biosimilar products. This framework was followed based on that previously established by the WHO in 2009. WHO’s biosimilar regulatory framework authorized globally accepted conditions for the introduction of safe, effective, and quality similar biotherapeutic products (SBPs). The main goal of WHO’s regulatory framework was to assist as well as ensure that local regulatory bodies followed the international standards of biotherapeutic production. Other countries gradually adopted these guidelines as their own, while few others authorized their own guidelines that were based on the existing models. In 2009, Japan and Korea released their own regulatory frameworks for biosimilars [26]. Countries, like Australia, took up the EU guidelines without any alterations. Malaysia and Singapore adjusted theirs to meet the standards set by the EMA guidelines. Countries, such as Brazil and Cuba, adopted WHO’s guidelines as a foundation for their own biosimilar regulations. Countries continue to set their own regulations that are based on the current trends, such as India, which released their guidelines later in 2012. The US was a late entrant in the biosimilar regulation pathway introduction, with their approval for biologics being made through the Public Health Service Act. The Biologics Price Competition and Innovation Act (BPCIA) was signed in 2010 as part of the Patient Protection and Affordable Care Act, which created a new licensure pathway for biosimilars. The BPCIA ensured the availability of drugs at affordable prices for the public, and bolstered innovation by companies producing originator biologics. From its first biosimilar approval in 2015 to 2017, four biosimilars have been sanctioned in the USA for the treatment of 23 indications [27]. Canada also issued their guidelines in 2010 under the approval of their federal authority, Health Canada [28]. The first anticancer mAb biosimilars were approved in Europe in 2017 [29,30]. By September 2017, a biosimilar of bevacizumab was authorized by the US FDA as the first anticancer biosimilar product for market approval in Europe [31].

**Figure 1:**
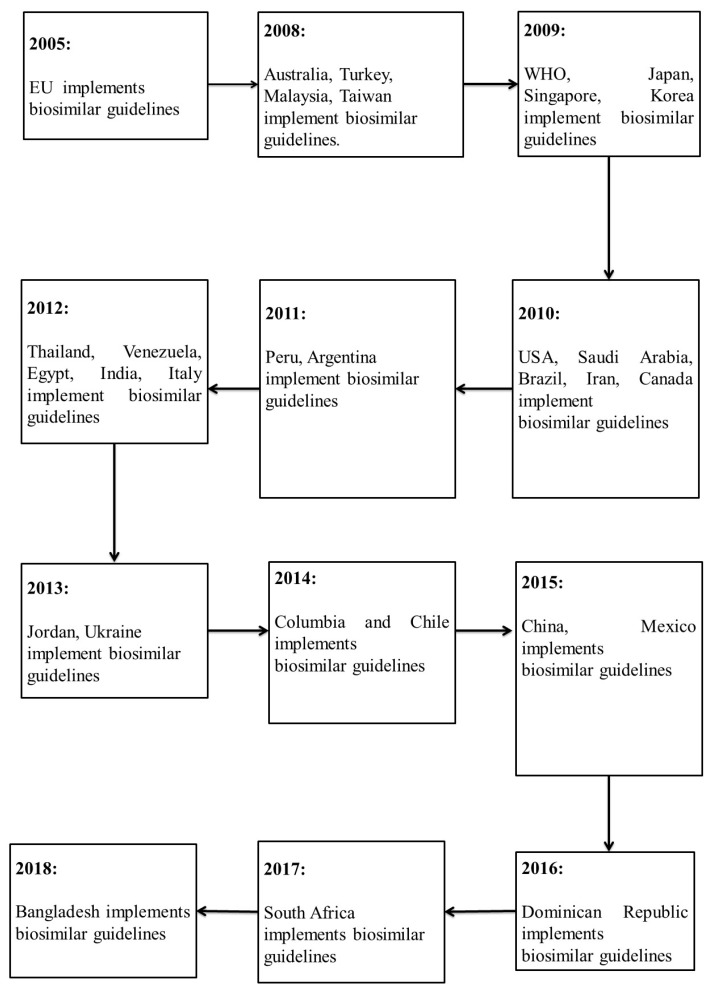
Progression of regulatory guidelines implementation for biosimilars from 2005 to 2018.

Figure 1, below, illustrates the approval of biosimilar regulations in various countries between 2004 and 2018.

#### Comparison of Biosimilar Regulatory Guidelines

The regulatory requirements for approval of biosimilars are generally consistent across the guidelines issued by the EMA, WHO and the FDA. Although there are minor differences, with some slight differences in terminology, all necessitate a stepwise approach to establish biosimilarity. These established regulatory pathways incorporate comparative assessments that involve analytical, non-clinical, and clinical studies. The regulations require head-to-head comparative studies for structural characterization, functional in vitro assays, pharmacokinetic and pharmacodynamic evaluations, and safety, efficacy, and immunogenicity assessments. Biosimilarity is demonstrated based on the totality of the evidence across all evaluations, with each step being supported by the preceding one of the process. Table 2 below shows a comparative overview between the regulatory guidelines of EMA, WHO, US FDA, BGTD, PMDA, and TGA [32].

As seen in the Table, the US FDA, BGTD, and PMDA carry out more stringent clinical trials for establishing biosimilarity through the inclusion of immunogenicity evaluation. Geologically, the biologics and biosimilars market are categorized into three divergent clusters—the US, other advanced economies (Europe, Japan, and Canada), and the pharma-emerging markets. The US accounts for a majority of global spending on biologics as well as plays a major role in driving biosimilar market potential. While the progressive economies possess the benefit of an established framework for biosimilars, to date the uptake of biosimilar drug products has been deliberate. On the other hand, the Europe biologics and biosimilars market still remains the most superior [33]. Sharp growth rates for biologics are currently observed within pharmerging markets, and this is where a large extent of the growth can be found.

Depending on whether the market is regulated, semi-regulated, or unregulated, biosimilars occupy distinctive positions. A number of issues are necessary to be considered by companies before they aim to set up production or market a product. Market and competitive demands for biosimilars vary from country to country, but they may be broadly categorized based on countries that are regulated (such as the US and EU) and those that are semi regulated (such as China and India) [34]. Manufacturers that aim to locally carry out their production must ensure that their product meets all of the requirements established by their national guidelines [35]. Manufacturers producing biosimilars for export to foreign markets must ensure that their products meet the guidelines that were established within the cluster that they are marketing to.

### 2.2. Approval Process for Biosimilars

The relatively contemporary introduction of biosimilars in the pharmaceutical therapy has urged the need for the development of step-by-step approval procedures for these agents before their commercialization. There is a unified priority for the maintenance of a high degree of similarity with the originator product. The approval process for biosimilar products involves stringent comparability exercises to test their quality, therapeutic efficacy, pharmacological activity, and safety of use. This process factors in evaluation strategies that begin from translating the product’s physical, biological, and chemical characteristics to testing its performance in clinical and non-clinical in vivo studies. The EMA holds credit for being the first drug regulatory authority to set such a framework. Other authorities, such as the WHO and the US FDA, are recognized for closely following suit [36]. The “totality of evidence” approach for approval that was established by the US FDA involves the collection of information from each phase of the biosimilar development. This is subsequently collated with the information that was obtained from that of the reference biologic. The principle establishes biosimilarity via a framework of data evaluation, which detects any significant differences and potential pharmaceutical impacts between the biosimilar and its originator product [37]. The safety of the biosimilar product is also assured through extensive comparibility studies. Similarly, WHO’s guidelines for biosimilars approval involve the appraisal of the biosimilar product’s physicochemical properties, in-vivo activity characterization, and pharmacological and toxicological results. These parameters are analysed via animal studies, comparative human pharmacokinetics (PK, concentration vs. time) and pharmacodynamics (PD, effect vs. time tests, clinical safety and tolerance, immunogenicity, and pharmacovigilance. The following case study provides an example of an approved biosimiar.

*The US. FDA has approved Ogivri (trastuzumab-dkst) as a biosimilar drug product to Herceptin (trastuzumab). The drug is employed in the treatment of patients with breast or metastatic stomach cancer (gastric or gastroesophageal, junction adenocarcinoma) whose tumours overexpress the HER2 gene (HER2+)* [38]*. Approval of the product was based on a review of comparibility evidence that evaluated data on its structural and functional characterisation, date from animal studies, clinical immunogenicity data, human pharmacokinetic and pharmacodynamic data, as well asclinical safety and effectiveness data. However, Ogivri is yet to be approved as an interchangeable product with its reference biologic* [39]*. Common side effects of Ogivri when used in treating HER2+ breast cancer include chills, nausea, rashes, headache, infection, congestive heart failure, fever, insomnia, coughing and diarrhea.*

*Case Study 1*

Biosimilar approval processes that were set by the most notable drug authorities worldwide have several similarities. However, certain factors, such as drug interchangeability, naming, and pharmacovigilance, may follow separate systems. The EMA regulatory approval framework does not provide any regulations on the basis of drug interchangeability [40]. This framework promotes the design of pharmacovigilance plans over post market monitoring. Several countries have set up their own individualized regulatory frameworks or adopted those that were issued by reputed and recognized authorities around the world. This complies with the acknowledgement that the approval schemes for generic medication could not be mirrored in the case of the more complex biosimilar drugs [41]. Certain countries, such as Russia, instate their own law for biosimilar approval. This law usually permits the registration of biodrugs under the definition of medicinal products possessing biologically active substances. Therefore, these countries do not have separate regulatory frameworks for biosimilar approval [42]. Biosimilars that were approved within these countries require complete clinical development programs, which are identical to those of the originator biologic products.

However, the concept of a separate biosimilar approval system remains alien or ambiguous to several remaining countries, particularly in third world nations. New systems are a tedious process to integrate within these nations. The limited affordability of healthcare products in such countries urges the need for the introduction of cheaper pharmaceutical products. Furthermore, the need for the development of pharmacovigilance programs to monitor the safety of approved biosimilars in clinical use has made the entire adoption process more exhaustive to poorer countries. Table A1 shows the timeline of biosimilars that were approved until 2019. Table A2 provides the details of these biosimilars that were approved by the EMA and the US FDA till 2019.

### 2.3. Extrapolation of Indications of Biosimilars

Biosimilar clinical studies demonstrate equivalent safety and efficacy in treating indications to those that are treated by the originator biologic drug are often adequate scientific justification for biosimilar approval. The mechanism of action, drug pharmacokinetics across patient populations, target receptors of the selected biosimilar must be identical across all range of diseases treated by its reference biologic [43]. Any variations in the safety or immunogenicity profile of the biosimilar have a major impact on regulatory decisions. The challenge here lies in the differences by which regulators evaluate evidence regarding biosimilarity. These differences can create uncertainty if they are divergent from extrapolation decisions of other regulatory bodies. Supporting clinical and non-clinical data on the biosimilar needs to be obtained in situations where comparability data is insufficient in assuring positive drug interchangeability. The employment of strong post marketing surveillance strategies would further ensure the success of indication extrapolation studies that were carried out on the biosimilar.

### 2.4. Future Perspectives of Biosimilar Evaluation

Improving access to biosimilars, as well as ensuring that they are utilized effectively in treatment, calls for a high degree of collaboration between multiple stakeholders who possess a distinct role in the regulatory pathway. With regulatory authorities wielding the pivotal responsibility of ensuring that only safe, high quality, and efficacious biosimilars attain commercialization, there is an increased need for the capacity of these authorities to be promoted. However, such a step poses to be particularly challenging, especially within countries possessing limited resources. In these cases, the establishment of regulatory procedures that improve the efficiency of the approval process could provide significant traction and benefit to biosimilar adoption. Approval could be facilitated via a collaborative review that was executed by other regulatory authorities or through a previous expert review. Furthermore, approvals of biosimilars that were obtained from regulatory authorities possessing the appropriate expertise could stand as a strong reference to these expert reviews [44]. The ‘Adaptive Designs for Clinical Trials of Drugs and Biologics’, a draft guidance that was recently made available by FDA for the industry, describes the important principles for designing, conducting, and reporting the results from an adaptive clinical trial to provide evidence of the effectiveness and safety of a drug or biologic. It has a variety of advantages over the non-adaptive designs, as the clinical trials can be adjusted to information not available when the trial began, and thus can be considered to be used for biosimilars in their evaluation for safety and effectiveness [45]. Regulatory authorities should be legalized to monitor the impact of biosimilars in public health systems in collaboration with other stakeholders. To assist, WHO has established global standards to ensure the quality, safety, and efficacy of biotherapeutics, including biosimilars, at all stages of their life-cycle [44]. These standards posit themselves as a strong basis for mutual recognition of regulatory oversight and for regulatory convergence at the global level.

## 3. Critical Challenges of Biologics and Biosimilars

Biologic and biosimilar drugs possess unique structural complexities as opposed to the relatively simpler configuration of small molecule generic drugs [1]. Major topics in the biosimilar formulary review include the evaluation of clinical properties (interchangeability (Appendix A6), immunogenicity, clinical data), information on product manufacture, product characteristics (naming, labeling), and institutional considerations (pharmacovigilance, patient education). The challenges that are associated with biologic and biosimilars have been highlighted, as follows:

(i) Manufacture.
(ii) Naming.
(iii) Packaging.
(iv) Labelling.
(v) Pricing.
(vi) Immunogenicity.
(vii) Pharmacovigilance.
(viii) Interchangeability.

### 3.1. Manufacture of Biologics and Biosimilars

**Figure 2:**
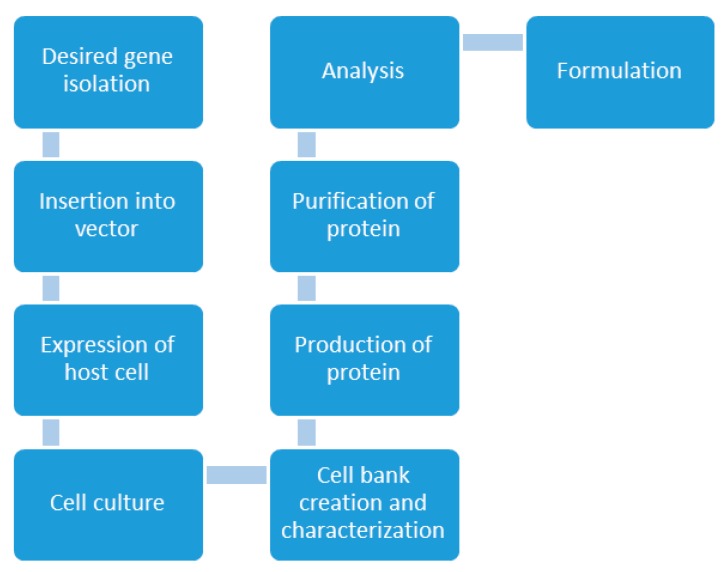
Conventional preparation process of biologics [13].

Unlike traditional chemical synthesis, the production of a biologic product from a living system does not follow an exact science as chemistry. Manufacturing biologics and biosimilars require the design of complex multistep processes utilizing mammalian and microbial cell cultures to manufacture therapeutic proteins [46]. “The process is the product” is a long existing paradigm of the biologic manufacturing process. This implies that any variations in the production process could significantly alter the product’s safety and efficacy profile. The current biologic manufacturing facilities combine both analytical and process development methods. This allows for the assessment of the scale up process while ensuring that it maintains the adequate productivity of a quality product [47]. Once the biologic has demonstrated sufficient safety and efficacy (Appendix A7), it then proceeded on to receiving regulatory agency and business feedback. The product is then finally approved and licensed. The steps in the preparation of a typical biologic are shown in Figure 2, below.

Impurities need to be constantly checked for and removed from the process due to the sensitivity of the process. During the biologic manufacturing process, the key contaminants are impurities in the host cell proteins (HCPs), cell debris, cell culture medium serum proteins, immunoglobulin affinity ligands, protein A or protein G affinity ligands, viruses, endotoxin, DNA, and non-protein cell wall constituents. A typical downstream process mainly consists of three stages: capture, intermediate purification, and polishing. These stages aid in the removal of soluble contaminants from crude feed, bulk impurities, and trace contaminants, respectively [48]. Other parameters also need to be closely monitored and maintained, such as pH, flow rate, temperature, purity and media. Manufacturers are required to maintain clean equipment and regulatory approved manufacturing methods [49]. The purification of biologics routinely involves chromatography (including affinity chromatography), filtration, and sterilizing filtration due to the complex nature of the proteomic pool.

The manufacture and testing of biological therapeutics have strong approved industry standards and manufacturers might tailor these standards to meet the specifications of the protein [50]. This includes developing individualized manufacturing methods, cell cultures, tests for release, and other specifications. In the context of biosimilars, these tests include a comparative analysis between the biosimilar and originator product to identify any degree of variation between the two [51]. When considering the lack or absence of information regarding the production process of the reference biological preparations, the manufacturing process of biosimilars needs to be carefully designed and closely monitored. This process comprises of selecting the appropriate originator biologic agent, detecting its critical molecular characteristics, and tailoring the process to match these traits. The manufacturing process is concluded by preclinical and clinical evaluation [52].

A reference of the biosimilar’s originator biologic license application does not need to be accessed by regulatory bodies to assess the biosimilar [53]. This is because up to date manufacturing standards and tests are required to be used by the biosimilar sponsor. Furthermore, the studies are compared with the data from already commercialized reference products. However, engineering of the process has to be done in the very early development stages within a narrow time frame. The need to commercialize the product as quickly as possible also entails less certainty of success. The design of a new process to replace an old one is highly costly and time consuming, which makes it tedious to scale up or scale down the manufacture of the biologic or biosimilar [49]. Manufacturers are often left to deal with the complex choice between having equipment sit idle or facing a supply shortage when redesigning a process to obtain a different volume of product.

The manufacturing process of biosimilars mandates the consideration of various important parameters [1,54]. Clinical and postmarketing studies have shown that appropriate target definition largely accounts for the safety and efficacy of the biosimilar end product [55]. The entire development process of a biosimilar drug is typically strategized via the following approach:

(i) Defining the target—This involves detecting any variability in the reference target molecule and any corresponding changes in qualitative properties of the drug. Choosing the type of biosimilar to be manufactured follows this.
(ii) Development based on target—The engineering process of the biosimilar is designed to match the criteria of the reference. These criteria include various factors, such as choice of cell line, biological processes, and development.
(iii) Similarity confirmation of the biosimilar—Degree of biosimilarity with the reference assessed via physical, chemical, and biological analyses of the biosimilar.
(iv) Regulatory authorization—Co-operating with relevant regulatory authorities to determine the minimum amount of clinical information required for biosimilarity approval.
(v) Clinical assessment—Conducting clinical trials to confirm biosimilarity and compiling any other information required for commercialization of the biosimilar.

#### Similarity Confirmation Process for Biosimilars

**Figure 3:**
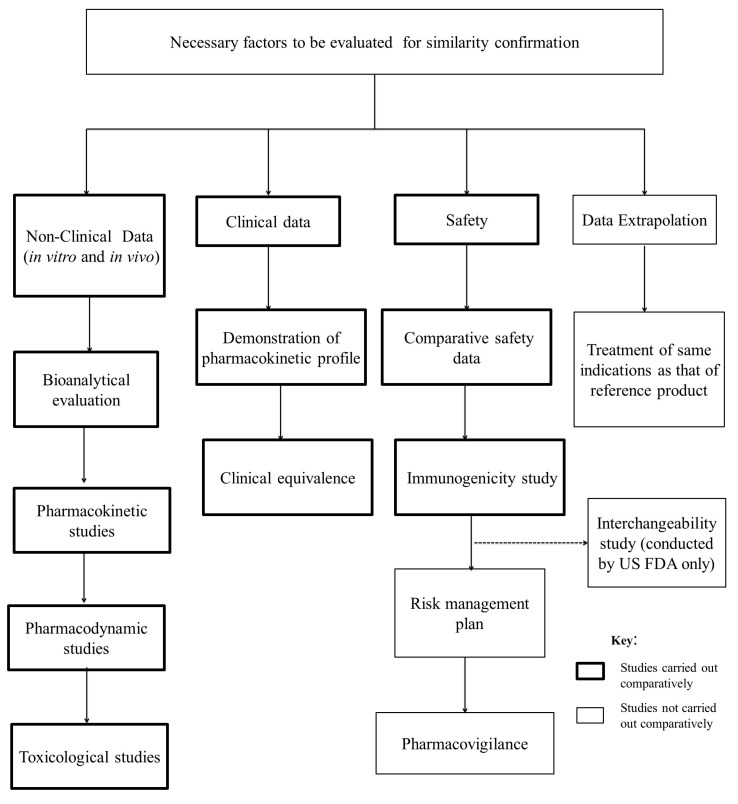
Factors to be evaluated for confirmation of similarity.

The complexity of biologics and their follow on counterparts has directed regulatory authorities to set a framework of scientific evaluation. This framework ensures that no differences that are present between biosimilars and their reference biologics have clinical consequences. The natural variability of these drugs has made them require a separate system of similarity confirmation from that of generic drugs. The similarity evaluation process of follow on biologics is summarized below (Figure 3).

Guidelines that are set by leading drug regulatory authorities worldwide dictate the need to maintain therapeutic equivalence of the biosimilar product with its reference. Any slight differences are strictly maintained within a narrow clinically accepted margin that is approved by the regulatory framework itself. The demonstration of biosimilarity revolves around several steps, starting from in vitro analytical testing and non-clinical comparative pharmacology testing, to toxicology, PK/PD studies, and clinical trials [56].

The non-clinical data that are required to assess the similarity between biosimilars and their originator drugs include the following:

(i) In-vivo studies: Animal models aid in the comparison in therapeutical activity of the biosimilar and its reference drug in the pharmaceutical form. The information obtained is valuable in deciding on the appropriateness of proceeding to further clinical trials.
(ii) Bioanalytical evaluation: Standard bioanalytical evaluation indicates the need for the product to show a high degree of analytical similitude with their reference. This comparison is done especially in terms of amino acid sequencing and folding, proportion of glycan and non-glycan components in their structure, stability profile, mechanisms of action, and purity. The similarity of the physicochemical and biological properties of biosimilar and reference preparations is demonstrated while using two or more orthogonal analytical methods. Ideal extensive bio analyses reveals negligible or no qualitative/quantitative variations in the functional and structural properties of the biosimilar compared to the originator biologic. Any areas of doubt, termed as “residual uncertainty”, in the efficacy profile of the biosimilar are investigated through PK/PD and immunogenicity studies [43].
(iii) Pharmacokinetic studies: Evaluation and comparison of the rate of drug clearance, effective drug absorption, total drug exposure over time, and drug half-life of the biosimilar product with its reference biologics aid in understanding the pharmacokinetic impact of the drug.
(iv) Pharmacodynamic studies: Data that were obtained from these studies aid in contrasting the reactivities of the biosimilar and its reference. This contrast is mainly done through the design of receptor binding studies and cell assays.
(v) Toxicological studies: The toxicity and immunogenicity between the biosimilar and its reference can be compared via repeat dosage toxicity studies. The data obtained are vital in assessing the safety of the follow on product and its tolerance within the body.

The safety guidelines issued for the biosimilar product are often centred on parameters that are also evaluated with regard to the originator biologic. It is acknowledged that these effects are difficult to predict from animal studies and they require further assessment through clinical studies. As a result, a large extension of data is also obtained from further clinical studies in a sufficiently large patient population for the monitoring of any therapeutically adverse event profiles [57].

Clinical studies include the comparison in human pharmacokinetic and pharmacodynamics profiles between the biosimilar and its originator biologic. It also includes the evaluation of biosimilar and biologic efficacy profiles with regard to the sensitive indications treated [58]. For biologics, these studies are categorized into the following three phases:

- Phase I: Pharmacokinetic and pharmacodynamics studies.
- Phase II: Dose Finding (DF) trials in order to measure the optimal biological dose.
- Phase III: Studies in all targeted indications and development of a risk-management plan.

For biosimilars, cost saving is often achieved in the elimination of expensive Phase II trials. Clinical evaluation studies are also reduced to those of a single representative indication.

**Figure 4:**
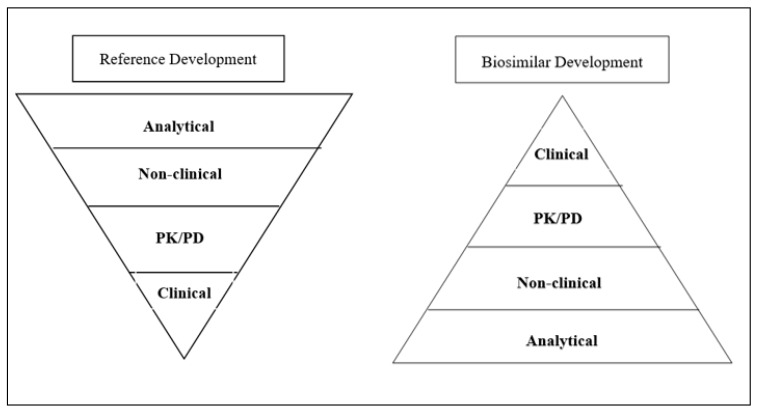
Comparison of the pre-marketing development processes of reference biologic and biosimilar.

Figure 4 represents the overall development processes of the reference biologic and the biosimilar product prior to commercialization.

The risk of immunogenicity is one of the most notable parameters in biological therapy. Regulatory guidelines for biologic and biosimilar drugs often address the need for risk assessment and mitigation plans to curtail any pre-clinical errors [59]. Bioanalytical assessment data may be vital in understanding the slight variations in safety and efficacy which may exist in the biosimilar product, although there is less necessity for an approved biosimilar to undergo separate licensing for other indications that are treated by the reference biologic. There is a risk of evaluating effectiveness, due to the fact that research is conducted in a small sample and a surrogate marker of the primary endpoint of effectiveness can be utilized. Extrapolation of indications needs to be assessed and approved individually by the regulatory authority for each individual indication. Any discrepancies in functionalities of the drug with regard to its mechanisms of action are investigated [60]. The extrapolation of the biosimilars to all clinical indications of the reference product is justified on the basis of experience following manufacturing changes made to protein therapeutics. Only rarely are any additional clinical studies required and, if necessary, would be conducted in a limited number of patients in a single indication. The ‘new’ product retains the approval of all clinical indications of the ‘old’ product. Thus, the same principles would apply to biosimilars [61]. The following case study provides an example of when additional research is mandated for extrapolation.

*In certain cases, challenges emerge in the extrapolation to all clinical indications for biosimilars. An example of this was seen when Health Canada decided to grant approval to the biosimilar anti-TNF antibody, Inflectra (infliximab) for only some of the clinical indications of the reference product, Remicade* [61]. *Inflectra was approved for use in rheumatoid arthritis, ankylosing spondylitis, psoriatic arthritis and plaque psoriasis, but not Crohn’s disease or ulcerative colitis. Health Canada proposed that differences of the biosimilar compared to the reference product in* in vitro *ADCC and binding to the FcγRIIIa receptor had a high chance of correlating with the mechanism of action in Crohn’s disease or ulcerative colitis. In 2013 however, the EMA came to a different conclusion on the same data* [61,62]. *Inflectra was approved as biosimilar for all eight of the clinical indications of the reference product. According to the EMA, the approval for all clinical indications should be viewed in the context of totality of data on analytical, preclinical, PK, PD and clinical information. Subsequent preliminary clinical studies sufficiently demonstrated the safety and efficacy of the biosimilar in paediatric Crohn’s disease, ulcerative colitis and inflammatory bowel disease* [63].

*Case Study 2*

### 3.2. Naming of Biologics and Biosimilars

It is essential that biologics, as well as products approved as biosimilars, be definitely identified and named to promote precision in writing and prescription filling [64]. Healthcare professionals need to be adequately educated in providing complete product names and accurate batch numbers to biosimilar medication to facilitate pharmacovigilance. While generic small molecule drugs tend to have the same names based on their active ingredients, the naming of biologics and biosimilars consists of identifying the variations in an already established product. This means it requires identification as a new biotherapeutic with a different name from that of the originator [65]. The conflict in naming arises when many users judge a biosimilar as a “new” product due to its distinct formulation, altered processing, varied trade name, or manufacturing company. Other users may deem the biosimilar product as similar enough to retain the same name, especially after their clinical comparability to the reference product has been established [66]. There is a necessity for healthcare providers to specify whether certain biosimilars will be deemed as the drug of preference or whether therapeutic interchangeability protocols will be allowed for biosimilars that have been approved as interchangeable [67]. The naming of biosimilars has been a bone of contention due to various innovator organizations preferring different INNs (A16) for biosimilars as opposed to their brand counterparts [68]. Individual regulatory regions tend to introduce their own biosimilar nomenclature schemes, while certain countries may conform to the regulation that is introduced by another region [69]. In Australia, the INN of a biosimilar ends with the suffix “sim” (indicating the word “similar”). This is followed with any letters the manufacturer of the product decides on. Japan follows a nomenclature system where the biosimilar INN is followed by the words “follow on” and the brand name having the letters “BS” incorporated into it [70].

In 2017, the US FDA introduced a regulatory framework where the NN (Nonproprietary Name) of a biosimilar should consist of a “core name” that is succeeded by a four-letter suffix. Normally, the USAN Council name is accepted for the reference product [71]. The World Health Organization’s final proposal for a biosimilar nomenclature scheme that was revealed in 2016 closely resembles the prior version of the FDA’s naming plan (addition of four random consonants as a suffix to the NN). The new proposal calls for the incorporation of a “biologic qualifier” (BQ) that comprises of four random consonants. This is accompanied by a non-mandatory two-digit checksum succeeding the NN of each biologic and biosimilar [72]. Table 3, below, shows a comparative overview for naming biosimilars between the regulatory guidelines of EMA, WHO, US FDA, BGTD, PMDA, and TGA [32].

### 3.3. Packaging of Biologics and Biosimilars

The packaging of biologics and their bio-generics poses several challenges to manufacturers due to their use of living organisms [73]. The proteins tend to be sensitive to metal ions (such as manganese, zinc, barium, and iron). This threat is pronounced by metals that leach from glass during steam sterilization (Appendix A8). Furthermore, tungsten oxide utilized in the glass syringe needle insertion method can induce protein aggregation and subsequent degradation of the drug. The packaging process is delicate, with the need to ensure that there are no organic extractable species (such as those that are found in polymers) or metals (such as those that are found in plastics and rubber), which are leached from the packaging materials [74]. These substances can significantly affect protein conformation and degradation, as well as present certain toxicity. Compatibility testing is essential in assessing the safety and quality of the product before commercialization.

### 3.4. Labelling of Biologics and Biosimilars

Labels on medicinal products are another important source of information that requires regulation in the case of biologics and biosimilars. This information is usually presented in the patient leaflet and the label on the outer product packaging [75]. Although it does not recapitulate the development and assessment history of the product, it provides information to clinicians, pharmacists, and patients with regard to the safe and effective use of the medication [76]. The appropriate labelling of biosimilars may allow for greater transparency. The clinical data would be useful in enabling the physician in making a more informed decision on which product is best for the patient [77]. Manufacturers strive to ensure that their biosimilar label is useful, fair, and informative in terms of purpose. In 2016, the US FDA released a draft stating the requirement for biosimilar labelling to include a description of the clinical data corroborating the safety and efficacy of the originator product [78]. The European Medicines Agency (EMA) follows a more science based conceptual approach for biosimilar labelling, and requires the label to be identical to the one that is utilized by the reference product [76].

### 3.5. Pricing of Biologics and Biosimilars

Biologics fall into the category of high costing pharmaceuticals, promoting issues that require addressing with regard to controlling healthcare costs and accessibility of the drugs to patients [79]. While certain guidelines promote competition and incentives for new drug pioneering (such as the BPCI), there is still a need to push prices down. Such a practice is currently being accomplished by generics, which have often taken the place of expensive branded drugs over the past years.

The introduction of biosimilars has signaled highly potential cost savings for patients that require treatment by biologics. Consequently, rather than generating the same full profile of nonclinical and clinical data as the reference product, a manufacturer that shows its proposed biosimilar product is highly similar to, and has no clinically meaningful differences from, the FDA-approved reference product may rely in part on FDA’s previous determination of safety and effectiveness for the reference product for approval. Hence, biosimilar manufacturers do not need to carry out as many expensive and lengthy clinical trials, which potentially leads to faster access to these products, as well as additional therapeutic options, and at reduced costs for patients [16]. An example of this can be seen in the following case study.

*Where massive price reductions are not a general expectation with biosimilars, small price reductions can still have a significant impact on healthcare costs. The list price of Basaglar in the US is set at a 15% discount as opposed to Sanofi’s originator insulin glargine, Lantus* [80]. *This allows for the saving of approximately US$56/month or US$1.86/day for a patient switching from Lantus to Basaglar. This has encouraged insurance companies and pharmacy benefit managers within the US to endorse Basaglar and provide exclusive formulary positioning to Basaglar over Lantus. While this may not be a problem for new patients, the issue of switching rears its ugly head when considering changing stable patients onto a biosimilar insulin. Although the US Food and Drug Administration has issued already issued a draft guidance concerning the interchangeability of biosimilars, it has, still not approved any biosimilar as interchangeable with its reference biologic* [71].

*Case Study 3*

According to the US FDA, manufacturers who are capable of demonstrating that their proposed biosimilars are highly similar to, and have no clinically meaningful differences from, the reference product are not required to generate the same full profile of nonclinical and clinical data as the reference product. Consequently, this results in biosimilar manufacturers not needing to conduct as many expensive and lengthy clinical trials, which potentially leads to faster access to these products, additional therapeutic options, and reduced costs for patients [16].

Biosimilars are required to be priced at a discount relative to their reference products, currently ≤30%, in Europe. This discount is likely to increase to 50% or more, eventually, in the US [81]. An example of this is the price comparison between the biosimilars Zarxio, Inflectra, and Renflexis and their reference products can be seen in Table 4 below [82,83,84]. Biosimilars must also compete with other biosimilars, with a high possibility of about 10 or more biosimilars being manufactured for each major reference product in major markets. Furthermore, they are also faced with competition from biobetters and other innovative products that target the same indications. Biosimilar manufacturers will be interested in protecting or growing their product portfolios and may bundle sales. The pricing of biosimilars may not always be rational (by conventional biopharma standards), with developers opting to low-ball their prices to capture market share.

As shown in the table, there is a significant price discount of 44% with Inflectra and a price discount of 35% with Renflexis when compared to their reference biologic Remicade. Furthermore, there is a 16-price discount with Zarxio as compared to its reference biologic Neupogen. Therefore, competition is also likely to rise as interchangeable biosimilars enter world markets, with these directly competing with the earlier branded biosimilars. Furthermore, since biosimilars are, by definition, highly similar to an originator’s reference product, there is little that a biosimilar company can do to differentiate such a product from the originator’s branded version other than offering it at a lower price [85]. A study in 2018 found that treatment with biosimilar regular human insulin (RHI) and insulin neutral protamine Hagedorn (NPH) could potentially cost ≤US$72 per year. With insulin analogues, this cost is expected to be below ≤US$133 per year [86]. Estimated biosimilar prices were markedly lower than the current prices for insulin analogues. Widespread availability at estimated prices may allow for substantial savings globally. Biosimilars are expected to reduce direct spending on biologic drugs by $54 billion between 2017 and 2026 [81].

The review of literature data shows that the differences between the price of biosimilars and their reference biologic product ranges between 15% and 30%. On the other hand, the difference for generics and originator drugs may be up to 80% [87,88]. A systematic review that was carried out by Tuna et al. to identify price difference between biotechnological reference products and biosimilars revealed that the variation differs across European countries between 0.51% and −38%. The review also stated that the expected cost savings are more than 10 billion dollars, regardless of the lower differences in comparison with the conventional and generic drugs [89]. Furthermore, a comparison of the prices of anti-TNF biosimilars between Canada and European countries identified a statistically significant difference—with the biosimilar price discount in Canada being greater (36%). This is followed by a price difference of 22% in Northern Europe and 18% in Southern Europe [90].

Biosimilars possess a strong impact on the competition in the biologics market. The use of biosimilars could significantly reduce healthcare expenditure on biological medicines within the EU national markets of Italy, France, Germany, Poland, Spain, Romania, the United Kingdom (UK), and Sweden by the year 2020 [91]. The biosimilars market predicts cost savings as much as $110 billion in Europe and the US by the year 2020 [92]. However, biosimilars are not perfect replacements for biologics. They still require expensive Phase III clinical trials (Appendix A9) and additional clinical studies before they can be introduced. Certain studies show that large Phase III clinical trials often do not contribute significant clinical equivalence information between the biosimilar and reference product [93].

It has been seen that several factors, such as manufacturing, development, pricing, clinician acceptance, and barriers to entry have caused large differences in the development of the biosimilar market opposed to that of the generic market [94]. Since biosimilars cannot be perfectly substituted with their originator biologics (as seen with generic and brand drugs), they enjoy more modest price discounts when compared to that of generics. Furthermore, manufacturers of biologics close to patent expiry tend to raise the price of their product in order to boost revenues before they encounter biosimilar competition [95]. The biologic Neupogen lost only about 10% of its market share once its biosimilar competitor Zarxio was inaugurated into the market [96].

### 3.6. Immunogenicity of Biologics and Biosimilars

Biosimilars pose to provide more affordable options for biological therapy in healthcare systems. They also aid in reallocating resources to other areas of patient care while maintaining equivalent safety, quality, and efficacy as that of their originator biologic. With increasing pharmaceutical manufacturers introducing biosimilars in the clinical landscape, these drugs are now recognized for treating a number of diseases [1]. The following case study provides an example of a disease area, which shows high potential for treatment by biosimilars.

*Rheumatoid Arthritis (RA) affects approximately 1% of the world’s population, primarily women aged between 30 and 55 years. This disease is indicated by the swelling, stiffness and pain in joints caused by the attack of the immune system on joint linings. RA is responsible for increasing disability to intolerable states among patients within five years of the disease diagnosis. RA patients also have a higher susceptibility to developing cardiovascular diseases, mental health problems and cardiovascular issues. Biosimilars have emerged as a new light of hope in RA treatment and are poised to provide new treatment options against the disease within the coming years* [97].

*Case Study 4*

Due to their very nature, biologics and biosimilars are capable of setting off immune responses in the human body. These responses may alter the safety or compromise the efficacy of the drug. Biologics are complex proteins and, by nature, are capable of initiating a humoral or cellular immune response. These can manifest in ways, such as anaphylaxis (Appendix A10), infusion reactions, loss of clinical efficacy, change in pharmacokinetics (Appendix A11), hypersensitivity (Appendix A12), or cross reaction with endogenous proteins [98,99]. This immunogenicity that has a potential to vary from that expressed by the reference product is influenced by patient, disease, and product related factors [100]. Immune feedback may affect the properties of the drug by promoting immune complex formation, alteration in the rate of clearance of the drug, and nullification of the therapeutic activity of the drug [101]. Patient related factors consist of variables, such as genetic background, pre-existing immunity, immune status, dosing schedule, and route of administration. On the other hand, product related elements, such as the manufacturing process, stability, and formulation, may also contribute to the likelihood of an immune response [102]. Furthermore, certain stabilizing agents and storage media can increase the potential of immunogenicity of a product. Such agents are particularly harmful when integrated with temperatures that are set beyond specification, and the product is roughly handled. Molecular modification processes, such as protein oxidation and aggregation, are also responsible for generating immunogenicity in the molecular structure. Identifying the root cause of the immune response is challenging, especially since several factors may interact to cause the problem [103]. Animals possessing the gene that codes for human insulin, interferons (Appendix A13), or tissue plasminogen (Appendix A14) activators tend to be effective anticipators of immunogenicity within patients. One of the more frequently used models for the prediction of immunogenicity in biosimilars is the immune tolerance exhibiting transgenic (Appendix A15) mouse [104]. However, models such as these require stronger quantitative validation in order to detect the subtle differences between the biosimilar and its reference drug. With the lack of forecasting ability of these models, clinical studies prove to be the more popular method for the establishment of biosimilar antibody induction. The results of animal immunogenicity studies are difficult to extrapolate to human. Therefore, the main assessment of the immunogenicity of biosimilar (as well as biological) preparations is carried out in clinical studies.

Many practicing clinicians are not completely aware of the utilities and risks of newly developed and commercialized biosimilars within the market [105]. This gap between science and practice needs to be bridged in order to keep up with current therapeutic trends and understand the interrelationship among their indications. This would also allow for healthcare professionals to compare the risks to benefits of treatment and improve clinical outcomes. Advocacy to relevant stakeholders, policy makers, and manufacturers is essential in order to ensure the access of safe, effective, and quality biosimilar medication to patients.

### 3.7. Pharmacovigilance of Biologics and Biosimilars

Pharmacovigilance is an essential aspect in the post approval process for biologics and, subsequently, biosimilars. This tends to be a challenge for such drugs, since the concurrent use of multiple medications by biologics taking patients makes it difficult to compile and evaluate an accurate safety profile from patients. Prescribers are often faced with the challenge of maintaining an appropriate monitoring system for which biologics have been prescribed for the patient undergoing therapy. The protein configuration of biologics and biosimilars also leave them vulnerable to molecular modifications in the body via a variation of biological pathways, which makes certain safety concerns that are associated with the drug only be detectable outside the timeframes of the controlled clinical trials carried out [46]. This strengthens the necessity for post approval safety monitoring and risk management of these drugs. Furthermore, the abbreviated licensure pathway of biosimilars places a bar on the amount of safety data available on the drugs. A strong pharmacovigilance strategy must compromise of safety specifications of the biologic or biosimilar drug, which summarizes its identified and potential risks, as well as identifies any region where there is a lack of sufficient information on the drug. These safety concerns should be addressed through well-defined activities and proposed actions with the added requirement of an evaluation of the potential for medication errors. Strict pharmacovigilance techniques are also necessary for identifying potential variation points in safety between biosimilars and their corresponding reference biologics. The following case study provides an example.

*Aimovig leads the line in a new class of drugs that are capable of inhibiting the calcitonin gene-related peptide, also known as anti-CGRPs* [106]*. The biopharmaceutical company Amgen (AMGN) is driven to tap Aimovig’s potential US market of 10 million patients through its indications in preventing migraines. The drug has already been approved and commercialized. Even though investors strongly believe that Aimovig is a promising asset, there have been some challenges. The drug is currently in competition with several other upcoming anti-CGRPs [such as Teva’s (TEVA) fremanezumab], oral anti-CGRPs, and Allergan’s (AGN) Botox. Furthermore, investors are concerned that the anti-CGRP class of medication will be restricted to just chronic migraine or patients that have failed Botox treatment. Initial post marketing surveillance data has been successful in reducing a few of these concerns* [107].

*Case Study 5*

Risk management strategies should be made available in the plan, which should highlight the effectiveness of the proposed activities as well as provide additional information, such as medication guide supplements and restriction of access [101]. The plan is designed to improve and expedite the biosimilar manufacturer’s faculty to obtain safety information of the product.

In this regard, a post-authorization safety study (PASS) needs to be carried out after a medicine has been authorized to obtain further information on a medicine’s safety, or to measure the effectiveness of risk-management measures. As an example, the European Medicines Agency’s Pharmacovigilance Risk Assessment Committee (PRAC) is responsible for assessing the protocols of imposed PASSs and for assessing their results [108]. PASSs can either be clinical trials or non-interventional studies, and the purpose of the information in PASSs is to evaluate the safety and benefit-risk profile of a medicine and support regulatory decision-making. They aim to:

- identify, characterize or quantify a safety hazard;
- confirm the safety profile of a medicine; or,
- measure the effectiveness of risk-management measures.

Table 5 below shows a comparative overview between the regulatory guidelines for pharmacovigilance between EMA, WHO, US FDA, BGTD, PMDA, and TGA [32].

### 3.8. Interchangeability of Biologics and Biosimilars

A biosimilar product can be considered to be interchangeable with its originator biologic if it is evaluated and approved to be capable of substitution with the reference product. This also includes meeting additional standards beyond bio-similarity [109]. The interchanging of medication addresses the medical practice of the physician exchanging the product that a patient receives with another drug product targeting the same therapy [110]. This switching is usually driven by a clinical imperative seeking an alternate suitable patient therapy. The need for alternate therapies are usually driven due to issues, such as efficacy or tolerability with the previous biotherapeutical product [111]. The US FDA identifies an interchangeable biosimilar as one that “can be expected to produce the same clinical result as the reference product in any given patient and, if the biologic is administered more than once to an individual, the risk in terms of safety or diminished efficacy of alternating or switching between the use of the biologic and the reference product is not greater than the risk of using the reference/product without such alternation or switch” [112]. No interchangeable biosimilars have been approved in the US yet, even though a recent guidance has been finalized by the US FDA [113]. It is important that the efficacy and safety risk of the biosimilar does not exceed the risk of administering the reference biologic without any substitution. This calls for stringent post marketing information on the biosimilar and crossover studies to determine its homogeneity with the reference product [114]. The interchangeability between biologics and biosimilars is not readily accepted in several countries for a number of arguments. This includes the doubt of value between the brand name biologic and generic drug and the concern over the authenticity of the biosimilars performance with regard to its differences from the biologic. Furthermore, the lack of differentiated regulations on the reference product, the biosimilar and their interchangeability hinders drug substitution acceptance. The following case study provides an example.

*The Brazilian health regulatory agency, Agência Nacional de Vigilância Sanitária (ANVISA), follows similar guidelines to interchangeability as that of the European Medicines Agency (EMA)* [115]. *While guidelines in the US connects interchangeability to substitution within pharmaceutical practice, interchangeability in Brazil constitutes medical practice within the scope of influence of physicians, patients, and Ministry of Health. Currently, ANVISA has not introduced any guidelines which specify the additional data or studies required to evaluate whether the biosimilar is interchangeable with its originator biologic. The present guidelines however dictate that the therapy directed towards the patient should not be switched multiple times between the biosimilar and comparator product for traceability reasons. Brazilian physician communities continue to push for an official interchangeability policy* [116].

*Case Study 6*

The lack of information that is available to the consumer with regard to interchangeability also discourages them from utilizing such biosimilars [117,118].

There are several considerations that need to be accounted for when interchanging biologic therapy with a biosimilar one. It is necessary to evaluate these factors in the perspective of the patient, disease, and product related factors. Potential risk factors have been summarized in Table 6, below.

## 4. Marketing Perspective of Biosimilars

Biosimilars are expected to play a huge role in the pharmaceutical market within the coming years. As more and more of these drugs are headed to be approved across a wider range of therapeutic areas, biosimilars have the potential to significantly boost treatment options in biological therapy. The worldwide market for biologics is expected to exceed $390 billion by the year 2020 from $46 billion in 2002. This will entail with it accounting for 28% of the global pharmaceutical market [11].

### 4.1. Opportunities of Biosimilars in Global Markets

One of the core objectives of biosimilar introduction is the promotion of healthcare cost savings, biological treatment attainability, and the subsequent improvement in patient outcomes. The development of biosimilars hopes to lead to more competition, and subsequently greater accessibility to cost-effective treatments. While biosimilar manufacture entails several challenges, they take lesser time to be developed, approximately five years less than that for an originator biologic [64].

Biosimilar manufacturers tend to develop their market strategy with the goal of improving the access of affordable biological healthcare to patients within sufficiently health conscious populations. Such manufacturers tend to target markets where their buyers are reasonably prosperous and their sales efforts cascade towards the strongest possible impact in the local populace. Therefore, the growth of biosimilars is often seen to accelerate within developed and recognized markets when compared to that in emerging markets of developing countries [119]. The availability of clinical safety and efficacy data has made the redevelopment of healthcare cost models possible. This allows for patients undergoing biosimilar treatment to enjoy more cost effective medication. There has been considerable international discussion on how to deal with biosimilars and other biological copies with regard to their regulatory aspects, especially in terms of their quality, safety, and efficacy evaluation. Identifying effective methods to distribute information among regulators is also of utmost importance [120].

The growth of the biosimilar market has seen key launches in several pharmaceutical market segments. These markets include those of human growth hormones (HGH), monoclonal antibodies (MAbs), erythropoietins (EPOs), insulins, human interferons (IFs), and granulocyte colony stimulating factors (G-CSFs). The introductions of these biosimilars have innovatively upgraded treatment strategies for a wide range of indications. The market for biologicals is primarily fuelled by factors, such as the rising demand for biological treatment of chronic diseases, such as diabetes and cancer. The following case study provides an example.

*Bevacizumab-awwb (Mvasi) received approval by the US FDA for the treatment of adult patients with certain cervical, colorectal brain, kidney, and lung cancers* [121]. *This biosimilar is a recombinant IgG1 monoclonal antibody that is capable of binding to vascular endothelial growth factor (VEGF), before inhibiting angiogenesis. Bevacimuzab is the first biosimilar approved in the US for anticancer therapy. Studies that were carried out include structural and functional characterization, data obtained through animal studies, immunogenicity studies, pharmacokinetic and pharmacodynamics profiles and safety and efficacy data.*

*Case Study 7*

However, it is constrained by the low affordability of these drug products, regulatory uncertainty, protocol for substitution, and complexity in production [122]. Table 7 shows how global markets for biosimilars can be categorized and compared:

Major factors to be considered in the introduction of a biosimilar product in the market:

(a) Cost of the biosimilar—Anticipation of how the market for the corresponding biosimilar will respond to a particular price is key for manufacturers of reference biologics. This enables them to determine the price at which their products should be before patent expiry [123]. Biosimilar manufacturers often target biologics possessing lower prices in order to maintain similar drug costs and levels of profit.
(b) History of the interchangeability of the biosimilar drug—Biosimilars that have a well-documented history of being substituted in place of their reference drug in therapy have a higher rate of acceptance for use in clinical settings and by patients [124]. Biosimilars for products, such as erythropoetins and granulocyte colony stimulating factors, have well-established use in replacement of their references. Therefore, these products have a higher preference by doctors and patients. On the other hand, biosimilars used to treat autoimmune diseases may require sufficient persuasion of clinics and patients for their adoption. This is usually attempted through the demonstration of their safety and interchangeability.
(c) Length of the therapy—Patients are more likely to vouch for those biosimilar treatment strategies that do not encompass a large period of time [125]. Patients tend to favour attaining significant cost savings and lowering the risk factor in being treated with a biosimilar product over the reference biologic.
(d) Patient involvement in choice of therapy—Programs that encourage patient participation on treatment decisions allow for greater confidence in selecting biosimilar options. These programs also allow manufacturers to differentiate their products based on the demands of the patient market. Such strategies require strong evidence of the safety and efficacy of the biosimilar drug. This is required to sufficiently convince patients that biosimilars are a safe, effective, and cost effective option in biologics treatment [126].

A lot of opportunities are present for emerging biosimilar markets where the majority of biologics that are prevalent in the market are non-originators [127]. Defined by IMS Health as “pharmemerging markets”, they consist of several markets (China, Algeria, Brazil, Argentina, India, Egypt, Colombia, Indonesia, Mexico, Turkey, Saudi Arabia, Pakistan Thailand, and Venezuela) [128]. Most of these economies have constituted their own biosimilar regulatory processes, although some of them are yet to be standardized. Their products are not considered as biosimilars, and they fall under the category of non-original biologics. Others draw on the framework that was set by the EU, with less stringent barriers with regard to acceptable clinical data limits and regulatory guidelines. Such a strategy enables local manufacturers within these countries to evenly compete with their international counterparts and boost the local production of biologics. This loose regulatory framework promotes a more rapid expansion of these pharmemerging markets and research into new areas of biosimilar development in the future. However, biologic drug therapy still remains out of reach to most patients within these emerging economies [129]. Furthermore, the drug products that are produced in these countries are not accepted in markets following the stringent guidelines set by WHO, EMA, and the US FDA. The major hurdles that are faced by biosimilar manufacturers are:

(a) Variation in regulations: There is high variability in regulations worldwide, regarding the approval of biosimilars. In Europe, the regulatory pathway is much clearer and more defined. On the other hand, in the US., there are stringent but very broad IP and patent laws, which may lead to a major obstacle in effective marketing leading to patient inaccessibility and increase in cost [130]. Certain countries attempt to produce biosimilars without undergoing the standardized regulatory evaluations in terms of the quality, safety, and efficacy. WHO developed a guideline in 2015 that provides a road-map for the regulatory assessments of such products. Under this guideline, these products are classified as non-original biologics since they have been registered without a comprehensive, head-to-head comparison with a reference biologic [131].
(b) Complexity of manufacture: The manufacturing process of biosimilars tend to be more tedious than that of the reference biologic [1]. This results in increased time, risks, and costs of productions pushing the market prices of these drugs to be higher than expected once they are commercialized.
(c) Greater competition: Biosimilar products face market competition from both “biobetters” (improved versions of the innovator biological molecule engineered in order to improve safety and efficacy) as well as competing brands of biosimilars [132]. Providing sufficient demonstration of the reliability and effectiveness of the biosimilar product is a time-consuming process. Manufacturers of biosimilars with long treatment plans face the burden of keeping their footing with their market rivals.
(d) The need for greater caution during drug substitution: Lack of clear interchangeability guidelines within pharmemerging economies lead to reluctance in physicians to prescribe a particular biosimilar over its reference. This can be resolved by the provision of sufficient assurance of the product’s therapeutic equivalence and safety.

### 4.2. Strategic Perspective for Biosimilar Market Entry

Biosimilars are viewed as a future source of value as well as an avenue of achieving healthcare cost savings without compromising on quality. The increasing need for lower healthcare costs in all pharmaceutical markets has seen a sharp increase in the demand for generics and biosimilar drug products [133]. Furthermore, biosimilars pose the opportunity of providing of a more sustainable healthcare system with more room for innovation. They also possess the continued assurance of biological healthcare to patients. A strong and clear strategy is required to unlock the potential of biosimilars within a pharmemerging economy. Well defined policies are required along every phase of the value chain, from the product clinical development to its commercialization. Until now, the manufacture of these drug products represents an ideal solution for meeting the high needs for patient access and affordability of biological treatment. A strong framework is essential to aid companies in capitalizing on the benefits of biosimilar market growth. Such a framework would also aid in overcoming market barriers, such as quality considerations, lack of technology and regulatory policies, and high costs of production. The benefits that are afforded from biosimilars can be evaluated from both the stances of retaining current value or generating new value [87]. Companies that are focused on the former are required to calculate, manage, and lower their risk of allocating resources and achieving cost efficiencies. On the other hand, companies with an eye for generating new value are required to evaluate their strategic fit and requirements for successful entry into the market. Consequently, they also need to be able to design a favourable market model for the product.

Several factors which have been recognized [134] as being vital for the entry and prevalence in the biosimilars market are as follows:

(a) capacity of the company to fund basic research and promote the development of clinical trials;
(b) accessibility to specific and reliable biosimilar development platforms;
(c) supporting services such as legal expertise and distribution channels to facilitate the entry of the product in the market;
(d) access to a global network of marketing representatives;
(e) lobbying with local regulatory authorities, governments and opinion leaders to allow for the approval of the biosimilar drug as a substitute of the reference product in therapy and to initiate competition between the biosimilar and its reference; and,
(f) experience in the manufacturing of biological and/or biosimilar products.

Certain enterprises are capable of engaging in joint venture and co-development strategies to facilitate the entry of biosimilars into the market. For this, they need to be located in countries that possess a high commercial potential for biosimilars as well as a strong competitive index that is based on the market’s biologics accessibility. Co-operation with multinational companies could allow the access of foreign expertise and manufacturing capabilities. This is facilitated through the outsourcing of activities, such as cell line development, manufacture of biologics and biosimilars, process scaling, and any required technology transfer. In return, manufacturers can enable strong government relations and access to tenders for any future biosimilar production plans within the country. Joint venture also promotes the access to a wider market and the subsequent affordability of branded biologics.

Licensing is a well-defined strategy for companies in countries that possess lesser commercial potential and yet high levels of competition within its markets. This enables multinational companies to obtain access to smaller markets, equipped with the already developed market strategies of local companies existing within the market. This also enables MNCs to now get access to both the market, which can afford their reference biologic drugs as well as those segments that can only afford the lower costing biosimilar versions in the local market.

Companies in countries that possess low competition but relatively high commercial potential have the greatest opportunity to introduce their own products. This provides them the opportunity to capture a larger proportion of market shares and control. This is a common situation in new pharmemerging markets where companies that commercialize their products earlier get an upper hand over their successors in the market.

Finally, companies localized in countries that have shortcomings in both commercial potential and competitive strength in its markets would have to resort to outsourcing. Outsourcing strategies would allow such companies to suitably benefit from the market opportunities of biosimilar growth. This also enables these companies to utilize the expertise and infrastructure of other companies, instead of investing in the development of their own internal capabilities or relying on those of their local counterparts.

Certain companies vouch for the simultaneous development and production of new generation reference biologic drugs, while operating on the growth opportunities of biosimilars. This gives them the added advantage of maintaining competition within the biosimilars segment of the market. It also expands on their commercial opportunities through biologics manufacturing. The promotion of both biosimilars and biologics can be challenging if the devised business models conflict with each other, but if well built, can be highly remunerative.

### 4.3. The Nocebo Effect of Biosimilars

The nocebo effect is a negative effect of a therapeutical treatment (pharmacological or non-pharmacological), which is a result of the patient’s perceived expectations. It is not the physiological consequence of the treatment itself. This effect has a negative impact on the treatment adherence rates in patients undergoing biosimilar treatment [135]. The true burden of this effect on biosimilar treatment is difficult to measure. However, it is important for clinicians and manufacturers to understand the patient related factors and the psychological mechanisms affecting nocebo responses to biosimilar treatment [136]. Educating patients on the side effects of the biosimilar to promote prescription transparency is a suitable strategy to minimize the nocebo response. Furthermore, clinicians are encouraged to build strong relationships with their patients. These aid in enabling confident shared decision making and information exchange. It is the responsibility of healthcare professionals to identify patients who are at a risk for the nocebo effect. Professionals are also required to discern patients’ perceived expectations during an adverse event and reassure them of their treatment if their nocebo response is excessive [137].

## 5. Conclusions

The problems of an aging population and the associated increase in the prevalence of chronic diseases are a matter of great concern worldwide. Biologics have been found to be successful in the treatment of many life-threatening chronic conditions. With the expiry of patent protection of these effective drugs, biosimilars have emerged as promising alternative treatment options. Like biologics, biosimilars are sensitive to both the inherent variability of the protein production, or expression, system, and to changes in manufacturing processes. Thus, no biosimilar can be scientifically or technically identical to the originator’s product. Therefore, establishing robust manufacturing and quality control procedures is essential in the biosimilar production process. With the continued rise in healthcare expenditure, biologics and the emergence of biosimilars thus represent a solution to improving patient accessibility to biotherapeutical therapy. However, for these drugs to have their maximal intended effect within the healthcare system, there is a need for healthcare professionals and patients to grow more familiar with them. The advent of a globally consistent approach to biosimilar approval and strong policies for biosimilar interchangeability promises to promote clinician and patient confidence in these drugs. Efforts to reduce biosimilar regulatory differences between individual regions would also aid in biosimilar adoption. The introduction of comparability studies and policies that reduce uncertainties in biosimilar interchangeability and immunogenicity could lead to substantial healthcare cost savings in the long run. The establishment of strong biosimilar pharmacovigilance systems could ensure continuous feedback on their therapeutical success. They could also maintain a patient centered approach to biosimilar availability. Biosimilar marketers are encouraged to positively frame and tailor information regarding their biosimilar drug products to patients, without discarding the ethical practices of informed consent. Overcoming these barriers would see pharmaceutical manufacturers nearing the finish line— with the goal of global biosimilar adoption.

## Figures and Tables

**Table 1:** Definition of biosimilars by major regulatory authorities worldwide.

Regulatory Guideline	Definition
European Medicines Agency (EMA)	A biologic medicinal product similar to another biologic medicine that has already been authorized for use
World Health Organization (WHO)	A biotherapeutic product that is similar in terms of quality, safety, and efficacy to an already licensed reference biotherapeutic product
United States Food & Drug Administration (US FDA)	A biologic product that is highly similar to the reference product notwithstanding minor differences in clinically inactive components and that there are no clinically meaningful differences between the biologic product and the reference product in terms of safety, purity, and potency of the product
Biologics and Genetic Therapies Directorate (BGTD)	A biologic drug that enters the market subsequent to a version previously authorized in Canada, with demonstrated similarity to a reference product
Pharmaceuticals and Medical Devices Agency (PMDA)	A biotechnological drug product developed by a different company, which is comparable with an approved biotechnology-derived product
Therapeutic Goods Administration (TGA)	A version of an already registered biologic medicine that has a demonstrable similarity in physicochemical, biologic, and immunological characteristics, efficacy, and safety, based on comprehensive comparability studies

**Table 2:** Comparative overview of Preclinical Data studies and Clinical Trials established by the regulatory guidelines of EMA, WHO, US FDA, BGTD, PMDA, and TGA.

Regulatory Guideline	Preclinical Data	Clinical Trials
EMA	Target binding; signal transduction, functional activity/viability of cells of relevance. If in vitro comparability is satisfactory, animal studies may not be required	Comparative PK/PD * studies followed by clinical efficacy and safety trials
WHO	Receptor-binding or cell-based assays; relevant biologic/PD activity, toxicity	Similar to EMA
US FDA	Structural analyses, functional assays; animal toxicity assessments, PK/PD, immunogenicity (unless determined not necessary by FDA)	Similar to EMA + Immunogenicity assessment studies
BGTD	Receptor-binding or cell-based assays; Animal PD and repeat-dose toxicity studies, and other relevant safety observations	Similar to EMA + Immunogenicity assessment studies
PMDA	Toxicity and pharmacologic assessments, PK, and local tolerance	Similar to EMA + Immunogenicity assessment studies
TGA	Similar to EMA	Similar to EMA

* PK (Pharmacokinetic); PD (Pharmacodynamic).

**Table 3:** Comparative overview for naming biosimilars between the regulatory guidelines of EMA, WHO, US FDA, BGTD, PMDA, and TGA.

Regulatory Guideline	Naming
EMA	Commercial name, appearance, and packaging should differ; INN * should be the same for related biosimilars
WHO	Changes are being considered to the current policy of using INN
US FDA	Draft guidance proposes that all biologics be given a four-letter suffix to the INN
BGTD	Not specified
PMDA	Non-proprietary name of the reference product followed by “BS” and an abbreviation to reference the manufacturer
TGA	Australian biologic name without a specific biosimilar identifier suffix (policy is under review)

* INN: see A16.

**Table 4:** Percentage cost comparison of a few biosimilars and their reference products.

INN	Name	Strength	Price	Cost Difference (%)
Filgrastim	Neupogen (Reference Biologic)	300 mg	$324.30	16.21%
	Zarxio (Corresponding Biosimilar)	300 mg	$275.66	
Filgrastim	Neupogen (Reference Biologic)	480 mg	$516.45	16.22%
	Zarxio (Corresponding Biosimilar)	480 mg	$438.98	
Infliximab	Remicade (Reference Biologic)	100 mg	$940/vial	44%
	Inflectra (Corresponding Biosimilar)	100 mg	$525/vial	
Infliximab	Remicade (Reference Biologic)	100 mg	$1167.82/vial	35%
	Renflexis (Corresponding Biosimilar)	100 mg	$753.39/vial	

**Table 5:** Comparative overview between the regulatory guidelines for pharmacovigilance between EMA, WHO, US FDA, BGTD, PMDA, and TGA.

Regulatory Guideline	Pharmacovigilance
EMA	Risk management pharmacovigilance plan must be submitted; clinical safety monitored closely after marketing authorization
WHO	Pharmacovigilance plan submitted with marketing authorization application; describe planned post-marketing activities
US FDA	Any risk evaluation and mitigation strategy for the reference product applies. Post-marketing studies or additional clinical trials could be mandated
BGTD	Risk management plan submitted prior to marketing authorization; periodic safety update reports. Serious adverse drug reactions reported within 15 days
PMDA	Post-authorization safety studies monitored on a continuous basis
TGA	Risk management plan outlining pharmacovigilance procedures to be implemented submitted with biosimilar application

**Table 6:** Risks and Considerations in Interchangeability.

Potential Risk Factor	Considerations	Sources of Evidence
Risk of product not being approved as per standards in global guidelines supporting biosimilar development	The presence of “non-comparable biologics” (NCBs) in certain parts of the world implies the existence of drugs that have not been directly compared with the reference product according to recognized guidelines and therefore may not meet global standards. Switching scenarios between these types of products and their reference product represent the highest level of uncertainty and risk to patient safety	Approved by Regulatory Authorities in accordance to WHO guidelines on Similar Biotherapeutic Products (SBPs)
Switching between the reference product and its biosimilar	Regulatory submissions for a biosimilar may sometimes consist of information on substituting the reference product for the biosimilar and/or vice versa. The quantity and type of this information will differ with each submission	For example, public assessment reports from Regulatory Authorities & scientific literature
Switching between biosimilars	The presence of clinical data directly comparing different biosimilars to the same reference biologic in the similar group of related products is very less likely to exist. This is not a mandate in regulatory filings. Switching between biosimilars represents an unknown, and one that harbours considerable uncertainty	There may be anecdotal or real world data available
Nature of the product	All biologics display a degree of immunogenicity, however, the nature and consequences of immunogenicity differ based on the product. Information regarding the reference biologic and biosimilar products may be of great assistance in this aspect. For biologics that are substitutes for a naturally-occurring hormone/cytokine/receptor, there is an increased risk of serious consequences, (e.g., if antibodies directed towards native proteins are produced)	For example, public evaluation reports from the related regulatory authorities and scientific literature
Route of administration and dosing device	Subcutaneous administration shows a greater degree of immunogenicity compared to intravenous administration. Usage of a different dosing device for the biosimilar may potentially increase the uncertainty as patients may be not be sufficiently familiar, when administering the product	Adequate labelling for biosimilar and reference products possessing proper dosing instructions
Extent and scope of post approval safety data	Where stringent systems for post approval safety monitoring of biologics including biosimilars exist, i.e., in jurisdictions compliant with WHO guidelines, such data may provide reassurance that the real-world use of the product does not result in any unexpected risks	Design and assessment of a risk management plan or evaluation of post marketing safety reports from related regulatory authorities. Overview of publications possessing review of safety data

**Table 7:** Categorization and comparison between global biosimilar markets.

Developed Markets	Emerging Markets
Most promoinent biosimilar markets e.g., EU, Japan, USA	Biosimilar markets with little or no presence e.g., China, Russia
Possess dedicated regulatory pathways	Tend to adopt regulatory pathways already set in developed markets
Possess stringent, abbreviated approval processes	Approval processes have less stringent comparability guidelines and therefore take lesser time
Physicians less open on reducing costs of therapy	Physicians more open on lowering the cost of therapy and increasing patient affordability

## Appendix A

A1: Antibody—An antibody is a protein produced by the body’s immune system when it detects harmful substances, called antigens. Examples of antigens include microorganisms (bacteria, fungi, parasites, and viruses) and chemicals. A2: Nanobody—A novel class of proprietary therapeutic proteins based on single-domain antibody fragments that contain the unique structural and functional properties of naturally-occurring heavy chain only antibodies. A3: Immunotherapy—The treatment of disease by inducing, enhancing, or suppressing an immune response. It is designed to elicit or amplify an immune response. A4: Immunoconjugate—A complex of an antibody and a toxic agent (as a drug) used to kill or destroy a targeted antigen (as a cancer cell). A5: Patent—A right, granted to an inventor, to exclude others from making, using, selling or importing an invention throughout the country without the inventor’s consent. A6: Interchangeability— In medicine, this refers to drugs that contains the same amount of the same active ingredients, possesses comparable pharmacokinetic properties, have the same clinically significant formulation characteristics, and is to be administered in the same way as the drug prescribed. A7: Efficacy—The maximum response achievable from an applied or dosed agent. It is the ability of a drug product to produce the desired effect. A8: Steam sterilization—Steam sterilization is a simple yet very effective decontamination method which is achieved by exposing products to saturated steam at high temperatures (121–134 °C). Product(s) are placed in a device called the autoclave and heated through pressurized steam to kill all microorganisms including spores. A9: Phase III clinical trials: These are randomized controlled multicenter trials on large patient groups (300–3000 or more depending upon the disease/medical condition studied) and are aimed at being the definitive assessment of how effective the drug is, in comparison with current ‘gold standard’ treatment. They involve a new treatment that’s worked well in a small number of patients with a certain disease. Doctors compare the treatment with the standard of care for that disease. The goal is to find out if the new treatment is better than standard treatment and/or with fewer side effects. A10: Anaphylaxis—Anaphylaxis is a potentially life threatening, severe allergic reaction that is rapid in onset and should always be treated as a medical emergency. Anaphylaxis occurs after exposure to an allergen (usually to foods, insects or medicines), to which a person is allergic. A11: Pharmacokinetics—Pharmacokinetics, sometimes described as what the body does to a drug, refers to the movement of drug into, through, and out of the body—the time course of its absorption, bioavailability, distribution, metabolism, and excretion. A12: Hypersensitivity—Hypersensitivity refers to excessive, undesirable (damaging, discomfort-producing and sometimes fatal) reactions produced by the normal immune system. A13: Interferon—Interferons are a family of naturally-occurring proteins that are made and secreted by cells of the immune system (for example, white blood cells, natural killer cells, fibroblasts, and epithelial cells). Three classes of interferons have been identified:

1. alpha,
2. beta, and
3. gamma.

Each class has many effects, though their effects overlap. Commercially available interferons are human interferons manufactured using recombinant DNA technology. The mechanism of action of interferon is complex and is not well understood. Interferons modulate the response of the immune system to viruses, bacteria, cancer, and other foreign substances that invade the body. Interferons do not directly kill viral or cancerous cells; they boost the immune system response and reduce the growth of cancer cells by regulating the action of several genes that control the secretion of numerous cellular proteins that affect growth. A14: Plasminogen—The inactive precursor of plasmin, occurring in plasma and converted to plasmin by the action of enzyme urokinase. A15: Transgenic—Transgenic is a term that describes an organism containing genes from another organism put into its genome through recombinant DNA techniques. An example of its usage is the term transgenic organism. A transgenic organism is one that contains a gene or genes which have been artificially inserted instead of the organism acquiring them through reproduction. A transgenic animal, for instance, would be an animal that underwent genetic engineering. It often contains material from at least one unrelated organism, e.g., from a virus, a plant, or from another animal. A16: INN - International Nonproprietary Names (INN) identify pharmaceutical substances or active pharmaceutical ingredients. Each INN is a unique name that is globally recognized and is public property. A nonproprietary name is also known as a generic name.

## Appendix B

**Table A1:** List of Biosimilars approved till 2019 by US FDA and EMA.

Name of Biosimilar Product	Approved by	Date of Approval	Manufacturer
Omnitrope	EMA	April 2006	Sandoz
Binocrit	EMA	August 2007	Sandoz
Epoetin alfa Hexal	EMA	August 2007	Hexal
Abseamed	EMA	August 2007	Medice
Silapo	EMA	December 2007	Stada
Retacrit	EMA	December 2007	Hospira UK
Ratiograstim	EMA	September 2008	Ratiopharm
Tevagrastim	EMA	September 2008	Teva
Biograstim	EMA	September 2008	CT Arzneimittel
Filgrastim ratiopharm	EMA	September 2008	Ratiopharm
Filgrastim hexal	EMA	February 2009	Hexal
Zarzio	EMA	February 2009	Sandoz
Nivestim	EMA	June 2010	Hospira UK
Remsima	EMA	September 2013	Celltrion
Ovaleap	EMA	September 2013	Teva
Grastofil	EMA	October 2013	Apobiologix
Bemfola	EMA	March 2014	Finox Biotech
Accofil	EMA	July 2014	Accord
Abasaglar	EMA	September 2014	Eli Lilly
Zarxio	US FDA	March 2015	Sandoz
Basaglar	US FDA	December 2015	Eli Lilly
Benepali	EMA	January 2016	Samsung Bioepis
Inflectra	US FDA	April 2016	Celltrion
Flixabi	EMA	May 2016	Samsung Bioepis
Inhixa	EMA	July 2016	Techdow Europe AB
Thorinane	EMA	July 2016	Pharmathen S.A
Erelzi	US FDA	August 2016	Sandoz
Amjevita	US FDA	September 2016	Amgen
Movymia	EMA	November 2016	Stada
Terrosa	EMA	November 2016	Gedeon Richter
Amgevita	EMA	January 2017	Amgen
Solymbic	EMA	March 2017	Amgen
Renflexis	US FDA	April 2017	Merck & Co. Inc.
Cyltezo	US FDA	April 2017	Boehringer Ingelheim
Truxima	EMA	August 2017	Celltrion
Mvasi	US FDA	September 2017	Genentech
Ogivri	US FDA	December 2017	Mylan
Ixifi	US FDA	December 2017	Pfizer Inc.
Retacrit	US FDA	May 2018	Pfizer Hospira
Fulphila	US FDA	June 2018	Mylan NV
Nivestym	US FDA	July 2018	Pfizer Inc.
Hyrimoz	US FDA	October 2018	Sandoz
Udenyca	US FDA	November 2018	Coherus BioSciences
Herzuma	US FDA	December 2018	Celltrion and Teva
Ontruzant	US FDA	January 2019	Samsung Bioepis
Trazimera	US FDA	March 2019	Pfizer Inc.
Eticovo	US FDA	April 2019	Samsung Bioepis

**Table A2:** Details of Biosimilars approved till 2019 by US FDA and EMA.

Name of Biosimilar Product	Reference Product	Class of Biological Medicine	INN	NN
Zarzio Zarxio	Neupogen	Granulocyte Colony Stimulating Factor (G-CSF)	Filgrastim	X filgrastim-sndz
Remsima Inflectra	Remicade	Tumor Necrosis Factor (TNF) alpha blocker	Infliximab	X infliximab-dyyb
Erelzi	Enbrel	Tumor Necrosis Factor (TNF) Žalpha blocker	Etanercept	etanercept-szzs
Amgevita Amjevita	Humira	Tumor Necrosis Factor (TNF) alpha blocker	Adalimumab	X adalimumab-atto
Renflexis Flixabi	Remicade	Tumor Necrosis Factor (TNF) alpha blocker	Infliximab	infliximab-abda X
Cyltezo	Humira	Tumor Necrosis Factor (TNF) alpha blocker	Adalimumab	adalimumab-adbm
Mvasi	Avastin	Monoclonal Antibody (MAb)	Bevacizumab	bevacizumab-awwb
Omnitrope	Genotropin	Growth Hormone (GH)	Somatotropin	X
Binocrit	Eprex	Erythropoietin (EPO)	Epoetin alpha	X
Epoetin alfa Hexal	Eprex	Erythropoietin (EPO)	Epoetin alpha	X
Abseamed	Eprex	Erythropoietin (EPO)	Epoetin alpha	X
Silapo	Eprex	Erythropoietin (EPO)	Epoetin zeta	X
Retacrit	Eprex	Erythropoietin (EPO)	Epoetin zeta	X
Ratiograstim	Neupogen	Granulocyte Colony Stimulating Factor (G-CSF)	Filgrastim	X
Tevagrastim	Neupogen	Granulocyte Colony Stimulating Factor (G-CSF)	Filgrastim	X
Biograstim	Neupogen	Granulocyte Colony Stimulating Factor (G-CSF)	Filgrastim	X
Filgrastim ratiopharm	Neupogen	Granulocyte Colony Stimulating Factor (G-CSF)	Filgrastim	X
Filgrastim hexal	Neupogen	Granulocyte Colony Stimulating Factor (G-CSF)	Filgrastim	X
Accofil	Neupogen	Granulocyte Colony Stimulating Factor (G-CSF)	Filgrastim	X
Nivestim	Neupogen	Granulocyte Colony Stimulating Factor (G-CSF)	Filgrastim	X
Ovaleap	Gonal-f	Follicle Stimulating Hormone (FSH)	Follitropin alpha	X
Grastofil	Neupogen	Granulocyte Colony Stimulating Factor (G-CSF)	Filgrastim	X
Bemfola	Gonal-f	Follicle Stimulating Hormone (FSH)	Follitropin alpha	X
Abasaglar Basaglar	Lantus	Human Insulin Hormone	Insulin glargine	X X
Benepali	Enbrel	Tumor Necrosis Factor (TNF) alpha blocker	Etanercept	X
Inhixa	Clexane	Low molecular weight heparin (Anticoagulant)	Enoxaparin Sodium	X
Thorinane	Clexane	Low molecular weight heparin (Anticoagulant)	Enoxaparin Sodium	X
Solymbic	Humira	Tumor Necrosis Factor (TNF) alpha blocker	Adalimumab	X
Truxima	MabThera	Monoclonal Antibody (MAb)	Rituximab	X
Movymia	Forsteo	Para-Thyroid Hormone (PTH)	Teriparatide	X
Terrosa	Forsteo	Para-Thyroid Hormone (PTH)	Teriparatide	X
Ogivri	Herceptin	Monoclonal Antibody (MAb)	Trastuzumab	trastuzumab-dkst
Ixifi	Remicade	Tumor Necrosis Factor (TNF) blocker	Infliximab	infliximab-qbtx
Fulphila	Neulasta	Leukocyte Growth Factor	Pegfilgrastim	pegfilgrastim-jmdb
Nivestym	Neupogen	Granulocyte Colony Stimulating Factor (G-CSF)	Filgrastim	filgrastim-aafi
Hyrimoz	Humira	Monoclonal Antibody (MAb)	Adalimumab	adalimumab-adaz
Udenyca	Neulasta	Leukocyte Growth Factor	Pegfilgrastim	pegfilgrastim-cbqv
Herzuma	Herceptin	Monoclonal Antibody (MAb)	Trastuzumab	trastuzumab-pkrb
Ontruzant	Herceptin	Monoclonal Antibody (MAb)	Trastuzumab	trastuzumab-dttb
Trazimera	Herceptin	Monoclonal Antibody (MAb)	Trastuzumab	trastuzumab-qyyp
Eticovo	Enbrel	Tumor Necrosis Factor (TNF) alpha blocker	Etanercept	etanercept-ykro
